# Joint modeling of longitudinal measures of pneumonia and time to convalescence among pneumonia patients: a comparison of separate and joint models

**DOI:** 10.1186/s41479-022-00101-5

**Published:** 2022-12-25

**Authors:** Sindu Azmeraw, Yenefenta Wube, Demeke Lakew

**Affiliations:** 1 Department of Statistics, Faculty of Natural and Computational Science, Woldia University, Woldia, Ethiopia; 2 Department of Statistics, Faculty of Natural and Computational Science, Bahir Dar University, Bahir Dar, Ethiopia

**Keywords:** Time to convalescence, Pneumonia, Longitudinal measure, Survival model, Separate model, Joint model

## Abstract

**Background:**

Globally, pneumonia is the leading cause of children under age five morbidity and mortality with 98% of deaths in developing countries.

**Objective:**

This study aimed to identify the determinants of longitudinal measures of pneumonia and time to convalescence or recovery of under five admitted pneumonia patients at Felege Hiwot Referral Hospital, Bahir Dar, Ethiopia.

**Methods:**

A prospective cohort study was conducted among a randomly selected sample of 101 pneumonia patients using simple random sampling who were on follow up from December 2019 to February 2020. A Linear mixed effect model were used for the longitudinal outcomes and joint model for modeling both longitudinal and time to event outcomes jointly respectively.

**Results:**

The significant values of shared parameters in the survival sub model shows that the use of joint modeling of multivariate longitudinal outcomes with the time to event outcome is the best model compared to separate models. The estimated values of the association parameters: − 0.297(*p*-value = 0.0021), − 0.121) (*p*-value = < 0.001) and 0.5452 (*p*-value = 0.006) indicates association of respiratory rate, pulse rate and oxygen saturation respectively with time to recovery. The significant values show that there is an evidence to say that there is a negative relationship between longitudinal measures of respiratory rate and pulse rate with time to recovery and there is positive relationship between longitudinal measures of oxygen saturation with time to recovery.

Variables age, birth order, dangerous signs, severity and visit time were significant factors on the longitudinal measure of pulse rate. The significant factors related to longitudinal measures of oxygen saturation were birth order, severity and visit. From this we can conclude that birth order, severity and visit were significant variables that simultaneously affect the longitudinal measures of respiratory rate, pulse rate and oxygen saturation of patients at 5% level of significance.

**Conclusion:**

Results of multivariate joint analysis shows that severity was significant variable that jointly affects the three longitudinal measures and time to recovery of pneumonia patients and we can conclude that patients with severe pneumonia have high values of respiratory rate and pulse rate as well as less amount of oxygen saturation and they need longer time to recover from the disease.

## Background

Pneumonia is described as the inflammation of parenchymal structures of the alveoli and the bronchioles (lungs) [1]. Community-acquired pneumonia (CAP) is an infection that begins outside the hospital and/or diagnosed within 48 hours after admission to the hospital. Whereas, hospital-acquired pneumonia occurs in more than 48 hours after admission and without any antecedent signs of infection at the time of hospital admission [2, 3].

Pneumonia is usually caused by infection with bacteria or viruses and bacteria are most common causes of (CAP) with Streococcus pneumonia isolated about 50% of cases. In children about 15% pneumonia cases a number of drug-resistant versions of the infections are more common, including drug resistant Streptococcus pneumonia and Methicillin-resistant Stapylococcus aurous [4, 5]. The burden of medical response to pneumonia has significant challenges. Besides drug resistance to the bacteria, comorbid conditions like Malaria, TB, Sickle cell anemia, HIV/AIDS and risk factors like lack of exclusive breast feeding, alcoholism, smoking etc. commonly appear in pneumonia patients which leads to define the severity and risk scores of the disease in which used for clinicians to make care self-site decision as in-patients or out-patients [6].

Estimates from the WHO suggest that pneumonia is responsible for 20% of deaths in the under-five age group, leading to 3 million deaths per year [7]. In Africa especially in sub-Saharan Africa, by 2013 pneumonia was the second leading cause of child mortality that accounts a million child death about 15.8% of total deaths in the region [8]. A report by UNECIF indicated that 132,000 under five children killed by pneumonia in Congo which is the second cause of death next to malaria [9, 10] and Kenya accounted the highest number of under-five mortality due to pneumonia which accounts about 16% of total deaths among 15 East Africa countries [11]. Pneumonia kills up to 5 million children under the age of 5 years annually in developing countries [12].

In Ethiopia, pneumonia is a leading single disease killing under five children and it contributes about 18% of all cases (370,000) of under five deaths compared to diseases like diarrhea, AIDS, malaria and measles every year [13, 14]. Under five pneumonia is commonly measured through physiologic parameters (temperature, pulse rate, blood pressure, respiratory rate, and oxygen saturation) and the performance of (TCS) is decided through the longitudinal measures of those parameters [15].

Several cross-sectional studies have used scoring systems to summarize the level of symptoms within a cohort at fixed time-points following CAP [16, 17]. However the understanding of which predictor affects length of hospital stay has been hampered by lack of longitudinal studies. Recent studies provide insight on the background and clinical predictors of mortality and survival of pneumonia patients among children aged under 5 years [18]. These studies did not consider the true and unobserved effects of longitudinal measures of physiologic parameters which correlates with recovery time to determine the length of hospital stay for under five pneumonia patients. In this study, joint model of multivariate linear mixed model and cox PH model was used to find significant factors of longitudinal measures of pneumonia (respiratory rate, pulse rate and oxygen saturation) and time to convalescence jointly.

## Methods

### Study area and period

The data for this study was collected from FHRH, Bahir Dar, Ethiopia from 12th December 2019 to 30th February 2020. Bahir Dar is the capital city of Amhara National Regional state. It is found in north western Ethiopia and is 565 km from Addis Ababa. This hospital serves as referral hospital for the people who came from different surrounding areas.

### Study design and sampling

A prospective cohort study design was conducted from 12th December 2019 to 30th February 2020. The study population was all selected under five pneumonia patients who were on treatment and follow up at FHRH from 12th December 2019 to 30th February 2020 and who full fill the inclusion criteria. The samples were the number of children bounded in the inclusion criteria with in the study period since the study is a case study carried out within 3 months follow up period. Therefore, all the confirmed cases of severe pneumonia described as per WHO criteria, aged from 2 to 59 months were included in the study. Hence, the final sample size estimated for this study was 101 patients. To select the study participants, simple random sampling method using table of random numbers was employed.

### Variables in the study

Three longitudinal outcomes (Respiratory Rate in bpm, Pulse Rate in bpm and Oxygen saturation in mm Hg) and a survival outcome (time to convalescence or recovery in hour) were considered as dependent variables in this study.

### Data collection procedure

The longitudinal and the survival data containing the socio demographic and home based information were collected using primary data collection method by face to face interview of their care givers using well-structured questionnaires. In addition, the data containing clinical information found from their charts were considered. Both primary and secondary data were collected by trained pediatrician and statistician.

### Eligibility criteria

The inclusion criteria was children 2–59 months of age with their care givers (mothers or not) and admitted at pediatric ward by community acquired pneumonia during the study period. Whereas the exclusion criterion was children admitted at the hospital by disease other than pneumonia, pneumonia patients below 2 months and above 59 months and patients with incomplete medical records.

### Data analysis

In this study, a longitudinal data on the three measures of pneumonia (RR, PR and OS), recovery time of under-five pneumonia patients for the survival data, and socio-demographic factors, home based factors, child nutritional status and child illnesses at the base line were considered. The data were coded, entered and edited using SPSS version 26 and the analysis was done using SAS 9.4 and R software and the statistical decision was made at 5% level of significance.

### Survival data analysis

Survival data analysis is a class of statistical method which used to analyze data in which the time(usually measured in days, weeks, months or years) until the event (usually death, disease incidence, relapse from remission, recovery) is of interest [19]. Cox proportional hazards model of the survival analysis was used to estimate the length of time to recover from pneumonia and to identify factors related to time to recovery [20].

The Cox model is defined as:

1 $$h\left(t,x,\beta \right)={h}_o(t)\exp \left({x}_{i1}{\beta}_1+{x}_{i2}{\beta}_2,\dots ..,{\beta}_p{x}_{ip}\right)$$

### Longitudinal data analysis

A longitudinal study is statistical analysis of an observational research method in which response variable is measured repeatedly over time and those measurements taken from the same subject are correlated [21]. Longitudinal response may arise when measurements taken on the same subject or when measurements taken on related subjects. In both cases, the responses are likely to be correlated [22].

### Linear mixed effects model

The random effects contains subject specific random effect and are directly used in modeling the random variation in the dependent variable at different levels of the data. Before considering the multivariate linear mixed model, it is better to identify the covariates which have significant effect on the mean change of RR, PR and oxygen saturation measurements over time using LMM [21].

Let *y*_*ijk*_ represent the *j*^*th*^ observation of the *k*^*th*^ outcome variable for the *i*^*th*^ subject, where:

$$\textrm{i}=1,2,\dots \dots 101,\textrm{j}=1,2,\dots \dots .{\textrm{n}}_{\textrm{i}}\ \textrm{and}\ \textrm{also},\textrm{K}=1,2,3.$$

$${\begin{array}{c}\ \\ {}\ \textrm{N}\end{array}}_{\textrm{k}}=\sum_{\textrm{i}=1}^{\textrm{n}}{\textrm{n}}_{\textrm{ik}},\textrm{N}={\textrm{N}}_1+{\textrm{N}}_2+{\textrm{N}}_3=\textrm{Total}\ \textrm{number}\ \textrm{of}\ \textrm{observations}.$$

The vector (y_1ik_ + y_2ik_ + . ……. . + y_nik_)^T^ represents the *n*_*ik*_ observations of the *k* response variable from the *i*^*th*^ subject and vector (y_1k_, y_2k_, …. . y_nik)_^T^ represent the *N*_*k*_ observation for the *k*^*th*^ response variable across all response variables and subjects, finally the vector (y_1_, y_2_, y_3_, ………y_n_)^T^ represents the observations across all response variables and subjects. In the context of modeling the response variables, the linear mixed effect model for each response variable of subject *i*, taken at time *t*, can be specified by [23].

2 $${\displaystyle \begin{array}{c}\ {\textrm{Y}}_{\textrm{k}}\left({\textrm{t}}_{\textrm{ij}}\right)={{\textrm{x}}_{\textrm{k}}}^{\textrm{T}}\left({\textrm{t}}_{\textrm{i}}\right){\upbeta}_{\textrm{k}}+{{\textrm{z}}_{\textrm{k}}}^{\textrm{T}}{\textrm{b}}_{\textrm{ik}}+{\upvarepsilon}_{\textrm{ik}}\\ {}\ {\textrm{y}}_{\textrm{ik}}\left(\textrm{t}\right)={\upmu}_{\textrm{ik}}\left(\textrm{t}\right)+{\upalpha}_{\textrm{ik}}\left(\textrm{t}\right)+{\textrm{b}}_{\textrm{ik}}\left(\textrm{t}\right)+{\upvarepsilon}_{\textrm{ik}}\\ {}\begin{array}{c}{\textrm{y}}_{\textrm{i}1}\left(\textrm{t}\right)={\upmu}_1\left(\textrm{t}\right)+{\upalpha}_{\textrm{i}1}+{\textrm{b}}_{\textrm{i}1}\left(\textrm{t}\right)+{\upvarepsilon}_{\textrm{i}1}\left(\textrm{t}\right)\\ {}\begin{array}{c}{\textrm{y}}_{\textrm{i}2}\left(\textrm{t}\right)={\upmu}_2\left(\textrm{t}\right)+{\upalpha}_{\textrm{i}2}+{\textrm{b}}_{\textrm{i}2}\left(\textrm{t}\right)+{\upvarepsilon}_{\textrm{i}2}\left(\textrm{t}\right)\\ {}{\textrm{y}}_{\textrm{i}3}\left(\textrm{t}\right)={\upmu}_3\left(\textrm{t}\right)+{\upalpha}_{\textrm{i}3}+{\textrm{b}}_{\textrm{i}3}\left(\textrm{t}\right)+{\upvarepsilon}_{\textrm{i}3}\left(\textrm{t}\right),\end{array}\end{array}\end{array}}$$

where

*μ*_*k*_(*t*) is the average evolution of the *k*^*th*^ response over time and it is a function of fixed effect. The subject specific random intercepts *α*_*ik*_ and slopes *b*_*ik*_(*t*) describe how the subject specific profiles deviate from the average profile for the *k*^*th*^ response.

### Joint modeling of multivariate longitudinal with time to event outcome

In this study three correlated and longitudinally measured response variables were considered which can be jointly modeled with time to event outcome. The separate and the joint models assume that the longitudinal sub model has the form similar to the conventional linear mixed effects model while the survival model in the joint model includes a latent association function *w*_*i*_(*t*) [24]. Maximum likelihood approach was used to estimate the parameters for both longitudinal and survival sub models.

### Ethical consideration

This study was carried out in the location where the approval was obtained from the ethical review committee of College of Health Sciences, Bahir Dar University, and permission for data collection was obtained from Felege Hiwot Specialized Referral Hospital Management. There were no risks due to participation in this research project, and the collected data were used only for this research purpose. The study compiled with the principles set forth in the Declaration of Helsinki (1964) and all of its subsequent amendments. The written informed consent was obtained for caregivers of each patient prior to the data collection and all information collected from each caregivers was treated with complete confidentiality.

## Results

The study revealed that, the median recovery time of pneumonia patients admitted at FHRH was 72 hours with minimum and maximum recovery time of 18 hours and 96 hours respectively. Out of the total sampled pneumonia patients, 90 (89.1%) were recovered from pneumonia. When we fit the cox proportional hazards model using the candidate variables: residence, birth order, age of mothers, education of mothers, danger signs, cooking place, comorbidity and severity were significant factors affecting time to recovery of pneumonia patients at 5% level of significance (Table 1).

**Table 1:** Cox proportional hazard results to determine the time of recovery among children under five pneumonia patients in Felege Hiwot Referral Hospital, Bahir Dar, Ethiopia

Variable	$\hat{\beta}$	HR	S.E($\hat{\beta}$)	Sig.
Age	0.16750	1.82345	0.315732	0.59574
Sex (female)	−1.21382	0.297058	0.977216	0.1099
Reference = no breastfeed breast feed				
Mixed	0.143399	1.154190	0.498162	0.77346
Exclusive	3.147100	1.158470	0.422553	0.0067 **
Residence (urban)	−0.056120	0.94530	0.016721	0.00128 ***
Birth order (first)	−1.34430	0.260800	0.355784	0.0001***
Age of mothers	0.12816	1.13601	0.025561	0.00001***
Education (literate)	1.352184	3.865861	0.462331	0.00345 **
Cooking place (inside living room)	−0.903495	0.405101	0.393565	0.02202*
Smoking (No)	0.988334	2.68585	0.445810	0.026622*
Comorbidity(No)	0.934444	2.54578	0.306529	0.00323**
Danger signs(no)	0.823067	2.27745	0.312194	0.00838**
Reference = non-sever				
Mild severs	−1.48279	0.227003	0.487705	0.00236 **
Sever	−4.78569	0.008348	0.733199	6.7e-11 ***

*HR* Hazard ratio, *S.E* Standard error, *CI* Confidence Interval

### Separate analysis of longitudinal data

In this study, three longitudinally measured response variables of pneumonia patients were considered. The linear mixed model was used for all the variables; pulse rate, respiratory rate and oxygen saturation of patients. The study was started by exploring the mean and variance structure of those longitudinally measured response variables. The three longitudinal measures of pneumonia were approximately measured every 6 h a day from admission up to hospital discharge of under-five pneumonia patients. All of 101 sampled under five admitted pneumonia patients were at risk of pneumonia up to the third visit time (t = 12 hour), this tells that, for this study, the minimum follow-up time at which the patient get the event of recovery was the third visit (t = 18 hours) and the number of patients getting the event increases, whereas the number of patients with at risk of pneumonia decreases through visit time.

The study also revealed that, the average values of RR and PR decrease, whereas the average values of oxygen saturation increase through the visit time. At the end of the follow up, the overall average values of RR, PR and Oxygen saturation were 50.55 bpm, 131.20 bpm and 90.18 mmHg with standard deviation of 12.55 bpm, 27.37 bpm and 6.11 mmHg respectively (Table 2).

**Table 2:** The descriptive statistics of RR, PR and Oxygen saturation at each follow-up

Visit time in hours	RR			PR			OX		
	Mean	Sd.	n	Mean	Sd	n	Mean	Sd.	n
0	54.5455	15.34247	101	136.5056	38.47912	101	86.788	9.1910	101
6	53.6661	14.42290	101	134.722	31.62821	101	88.807	6.2838	101
12	52.7667	11.37633	101	133.2215	28.47834	101	88.773	5.3050	101
18	51.2228	12.25312	100	132.2531	25.56966	100	90.023	5.4669	100
24	50.9760	13.50433	94	132.0511	28.43025	94	90.300	6.2053	94
30	50.5668	12.70948	81	131.8674	25.28053	81	90.232	6.4997	81
36	50.3937	12.20908	72	131.1632	25.35005	72	90.929	4.3395	72
42	50.2008	12.28492	64	131.2676	27.74004	64	91.110	5.3345	64
48	50.0577	11.86685	56	130.5855	24.48126	56	91.131	3.7086	56
54	49.9998	11.59709	52	130.5568	19.14048	52	91.327	5.0965	52
60	49.7596	8.32274	45	130.3284	24.80085	45	91.953	4.0997	45
66	49.2445	9.97147	40	129.8560	25.22296	40	90.745	6.8530	40
72	49.1604	11.61746	36	128.9091	20.70669	36	92.222	4.3432	36
78	49.0624	13.12481	30	126.8290	21.21595	30	91.006	3.6403	30
84	48.7009	8.18892	23	127.3923	18.56895	23	91.110	5.1941	23
90	48.1520	12.19470	21	126.8291	12.85806	21	93.316	5.2921	21
96	47.8304	10.72720	11	119.8127	22.80112	11	93.636	5.5906	11
Over all	50.5503	12.55158	1028	131.2031	27.37457	1028	90.181	6.1137	1028

*RR* Respiratory rate, *PR* Pulse rate, *OX* Oxygen saturation, *s.d* Standard deviation, *n* number of patients

**Fig. 1:**
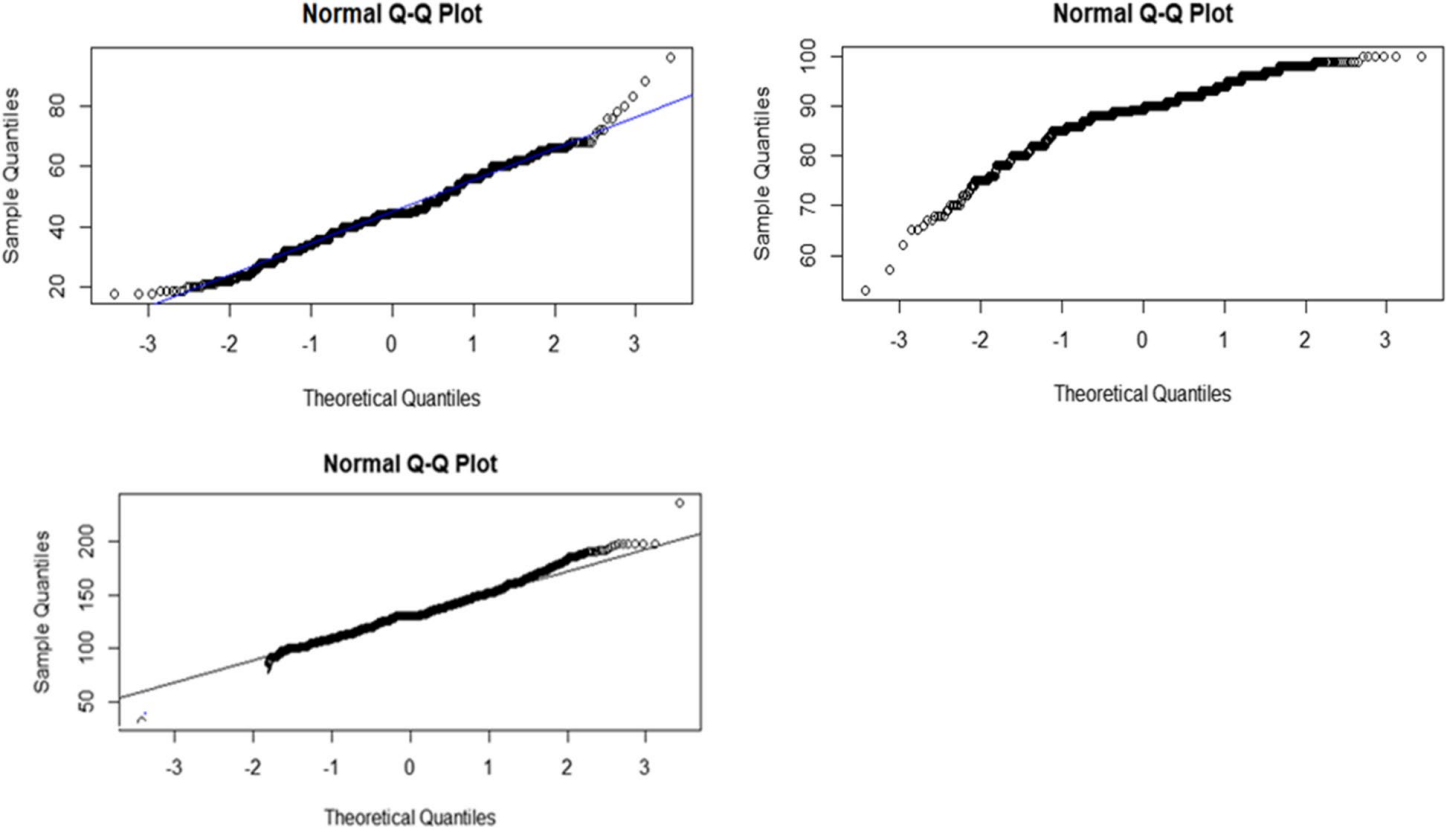
Normal QQ plots of RR, PR and oxygen saturation

Checking assumptions of the data is the first step in analyzing longitudinal data. Normal QQ plots in Fig. 1 shows that, the data for the three longitudinal outcomes were approximately normally distributed and then it is better to proceed to the next steps of the analysis.

### Multivariate analysis of longitudinal data

MLMM was fitted using three longitudinal measures of pneumonia (RR, PR and oxygen saturation) for under-five admitted pneumonia patients (Table 3). At 5% level of significance; marital status of mothers, smoking exposure of patients, breast feeding, severity, cooking place, comorbidity and visit time were significant factors related with longitudinal measures of RR. Age, residence, birth order, comorbidity, danger signs, vaccination, severity and visit time were significantly related with longitudinal measures of pulse rate. The variables that significantly related with longitudinal measures of oxygen saturation were; age at the base line, residence, comorbidity, danger signs, age of mothers, severity and visit time. Variables; severity, visit time and comorbidity were simultaneously associated with longitudinal measures of RR, PR and Oxygen saturation of patients.

**Table 3:** Results of multivariate analysis of longitudinal data for under-five pneumonia patients

MLMM for multivariate longitudinal data							
parameter		For RR		For PR		For OX	
		$\hat{\beta}(s.e)$	*p*-value	$\hat{\beta}(s.e)$	*p*-value	$\hat{\beta}(s.e)$	*p*-value
Intercept		48.86458 (3.38)	< 0.0001	162.1041 (9.22)	< 0.0001	92.97 (1.66)	< 0.0001
Age				−1.2573 (0.18)	< 0.0001	0.11718 (0.23)	< 0.0001
Residence(urban)		3.403193 (1.74)	0.0553	1.829784 (0.03)	< 0.0001	−1.4103 (0.63)	0.02568
Birth order(first)		2.916760 (1.95)	0.1507	9.202489 (4.12)	0.02560	−0.6652 (2.81)	0.8133
Reference = single							
Married		−3.91243 (2.21)	0.0773			3.5227 (1.19)	0.2497
Widowed		−1.2661 (2.51)	0.3360			1.3268 (0.82)	0.1064
Divorced		−1.11105 (2.91)	0.7037			1.3163 (3.62)	0.7167
Smoking(no)		−4.52168 (2.28)	0.0481				
Reference = no breast feed							
Mixed		−0.60634 (2.31)	0.7943				
exclusive		−1.6582 (0.35)	< 0.001				
Comorbidity(no)		−2.51715 (1.23)	0.04090	−3.76181 (1.80)	0.0367	1.40518 (0.62)	0.0236
reference = non-sever							
Mild sever		4.124048 (4.12)	0.0590	3.439812 (5.21)	0.5105	−2.8647 (1.04)	0.0058
Sever		9.658019 (4.76)	0.042	6.942920 (3.68)	0.024	−3.9492 (0.79)	< 0.001
Age of mothers		−0.8665 (1.18)	0.4628	−0.3784 (3.18)	0.9054	0.23458 (0.05)	< 0.0001
Visit		−1.06730 (0.07)	< 0.0001	−0.14986 (0.05)	0.0026	0.15926 (0.06)	0.0141
Danger signs(no)		−9.84996 (4.20)	0.0189	−7.64896 (3.69)	0.0382	1.70764 (0.72)	0.0177
Vaccination(vaccinated)				−11.5939 (5.61)	0.0389	0.5175 (1.01)	0.6310
Cooking place(inside living room)		3.12894 (1.63)	0.0460				
Education of mothers(literate)		−1.6479 (2.08)	0.4289			0.6979 (0.39)	0.9229
Random effect variance covariance matrix for MLMM							
Variance components		RR		PR		OX	
		Intercept	Slope	Intercept	Slope	intercept	slope
RR	Intercept	89.583	0.093	66.208	0.768	−1.358	0.0672
	Slope	0.093	0.062	−0.7034	0.356	0.110	−0.032
PR	Intercept	66.208	−0.703	210.99	−2.805	−5.201	0.249
	Slope	0.768	0.356	−2.805	0.821	0.401	−0.253
OX	Intercept	−1.358	0.110	−5.201	0.401	11.937	0.122
	slope	0.067	−0.032	0.249	−0.253	0.122	0.315
Sd.		12.305	1.141	22.719	1.101	2.001	0.561
Residual standard errors							
sigma2_1		8.6201					
sigma2_2		13.931					
sigma2_3		6.9933					

*RR* Respiratory rate, *PR* Pulse rate, *OX* Oxygen saturation, *MLMM* Multi variate linear mixed model

The random part of MLMM shows the variance and covariance between rate of change and baseline values for the three longitudinal measures of pneumonia (RR, PR and oxygen saturation) were significantly different from zero which tells the existence of a relationship between a patients baseline standing between outcomes, rate of change between outcomes as well as, between baseline standing of one outcome and rate of change of the other outcome through follow-up time.

### Joint modeling of multivariate longitudinal data and survival data

In the previous sections; determinants of the multivariate longitudinal measures of pneumonia as well as determinants of time to recovery of under-five admitted pneumonia patients were identified. The results of joint model analysis for multivariate longitudinal and survival data found in the Table 4, contains multivariate longitudinal and survival sub models. In the random part of MLMM, estimates of variance and covariance were different from zero, shows the existence of correlation between intercepts of outcomes, between rate of changes of outcomes and correlation between rate of change and baseline values of the three longitudinal measures of pneumonia (RR, PR and oxygen saturation).

**Table 4:** Results of joint model of multivariate longitudinal model and cox PH model

Longitudinal sub model							
Parameter(fixed effects)		RR		PR		OX	
		$\hat{\beta}(s.e)$	*p*-value	$\hat{\beta}(s.e)$	*p*-value	$\hat{\beta}(s.e)$	*p*-value
Intercept		47.26 (1.367)	< 0.0001	146.74 (5.99)	< 0.0001	87.2967 (0.236)	< 0.0001
Age		−0.371 (0.137)	0.0061	−1.012 (0.348)	0.0037		
Residence(urban)		1.700 (0.79)	0.0328	1.258 (0.4553)	0.0056	−1.001 (0.480)	0.0361
Birth order(first)		1.595 (0.66)	0.016	3.087 (0.0372)	< 0.0001	−1.715 (0.638)	0.007
Smoking(no)		−2.266 (0.82)	0.006				
Comorbidity(no)		−3.976 (1.96)	0.0416	−3.642 (1.0265)	0.0003	2.335 (1.0592)	0.0251
reference = non sever							
Mild sever		2.467 (3.18)	0.38	1.019 (1.2645)	0.9417	−0.006 (0.863)	0.5086
Sever		5.459 (0.86)	< 0.001	1.299 (0.0872)	< 0.0001	−1.032 (0.068)	< 0.0001
Age of mothers				−0.127 (0.655)	0.846		
Visit		−0.195 (0.05)	0.002	−0.160 (0.0766)	0.0360	0.901 (0.470)	0.026
Vaccination(vaccinated)				−8.593 (0.611)	< 0.0001	0.517 (1.01)	0.6310
Reference = no breast feed							
Mixed		−0.406 (1.31)	0.7943				
exclusive		−1.851 (0.265)	< 0.001				
Reference = single							
Married		−1.912 (0.21)	0.226			3.522 (1.19)	0.2497
Widowed		−1.266 (0.51)	0.3360			1.326 (0.82)	0.1064
Divorced		−1.111 (1.91)	0.7037			1.316 (3.62)	0.7167
Danger signs(no)						0.579 (1.4803)	0.6956
Cooking place(inside living room)						−2.105 (0.9823)	0.032
Education of mothers(literate)						2.704 (1.7495)	0.0106
Random effect variance covariance matrix for MV longitudinal sub model							
		RR		PR		OX	
		Intercept	Slope	Intercept	Slope	intercept	slope
RR	Intercept	85.203	10.082	65.251	0.798	−1.888	0.057
	Slope	0.082	0.021	−0.007	0.001	0.110	−0.080
PR	Intercept	65.251	−0.007	191.990	−2.801	−5.802	0.247
	Slope	0.798	0.001	−2.801	0.035	0.401	−0.071
OX	Intercept	−1.888	0.010	−5.802	0.401	10.901	−0.168
	slope	0.057	−0.080	0.247	−0.071	−0.168	0.023
Sd.		9.464	0.147	20.799	0.187	3.307	0.153
Residual standard errors							
sigma2_1		8.0701					
sigma2_2		10.7369					
sigma2_3		5.2133					
Survival sub model							
parameter		$\hat{\beta}(s.e)$		Hazard ratio		*p*-value	
Sex(female)		−0.3733 (0.5905)		0.681		0.5273	
Residence(urban)		−0.604 (0.275)		0.547		0.028	
Cook place(inside)		− 0.027 (0.559)		0.973		0.9604	
Comorbidity(no)		0.831 (0.400)		2.296		0.038	
Birth order(first)		−1.258 (0.120)		0.284		< 0.001	
Age of mothers		0.9011 (0.470)		2.462		0.026	
Reference = no breast feed							
Mixed		1.3360 (0.971)		3.803		0.169	
Exclusive		1.4011 (0.241)		4.060		< 0.0001	
Reference = non-sever							
Mild sever		−1.751 (0.692)		0.173		0.1205	
Sever		−1.581 (0.5164)		0.206		0.0022	
γ _1		−0.297 (0.041)		0.743		0.0021	
γ _2		− 0.121 (0.034)		0.886		< 0.001	
γ _3		0.5452 (0.2007)		1.725		0.006	

Based results of Table 4, the average RR, PR and oxygen saturation of under-five pneumonia patients admitted at FHRH were 47.2660 bpm, 146.7431 bpm and 87.29 mmHg respectively when all categories are at their reference group. As age of patients increased by 1 month, the average RR and PR were significantly decreased by 0.38 bpm and 1.01 bpm respectively. Whereas, age was not a predictor of oxygen saturation. Coming from urban residence increases the average RR and PR by 1.70 bpm and 1.26 bpm respectively, whereas it lowers the average oxygen saturation by1.01 mmHg as compared with rural residency, keeping other variables constant. Being first child significantly rises the average RR and PR by 1.59 bpm and 3.09 bpm respectively; whereas it lowers the average oxygen saturation by 1.72 mmHg as compared with being second or above child; other variables held constant. Being non-exposed by smoking lowers the average RR by 2.27 bpm as compared with patients exposed by smoking; keeping other variables constant, but had no information about PR and oxygen saturation.

Being non-comorbid significantly lowers the average RR and PR by 3.98 bpm and 3.64 bpm respectively, while it rises the average oxygen saturation by 2.33 mmHg as compared with being comorbid, keeping other variables constant. Having sever pneumonia at the baseline increases the average values of RR and PR by 5.46 bpm and 1.30 bpm respectively, whereas it lowers the average oxygen saturation by 1.03 mmHg as compared with those having non-sever pneumonia, other factors held constant. Having literate mother increases the average oxygen saturation by 2.70 mmHg as compared with those from illetrate mothers, held other variables as constant. Cooking food inside the living room lowers the average oxygen saturation by 2.11 mmHg as compared with those whose parents cook their food out of living room, keeping remaining factors constant. A unit increase in the number of visits lowers the average RR and PR by 0.19 bpm and 0.16 bpm respectively, whereas it rises the average oxygen saturation by 0.90 mmHg, keeping other predictors constant.

Getting vaccination lowers the average PR by 8.59 bpm as compared with unvaccinated by remaining other variables constant. Feeding exclusive breast within first 6 months decreases the average RR by 1.85 bpm as compared with no breast feeding. The estimated hazard ratio of patients from urban area relative to patients from rural area was 0.61 indicates, patients from urban residence were 0.547 times less likely to recover from pneumonia than patients from rural residence, other variables held constant. Patients without comorbidity were about 2.296 times more likely to experience the event of recovery compared to patients without comorbidity. Patients at the first birth were 0.284 times less likely to get the chance of recovery compared to patients at the second and above births, keeping other variables constant. As age of mothers increase by 1 year, experiencing the event of recovery increases about 2.462 times, other variables held constant. Exclusively breast feed patients within first 6 months of life were about 4.06 times more likely to get recovery as compared with patients having no breast feed.

Patients with severe pneumonia were about 0.206 times less likely to experience the event of recovery compared to patients with non-sever pneumonia, keeping other variables constant. The estimated values of association parameters γ_1 = − 0.297 (*p*-value = 0.0021), γ_2 = − 0.121 (*p*-value< 0.001) and γ_3 = 0.545 (*p*-value = 0.006) indicates; RR and PR were negatively associated with time to recovery, whereas oxygen saturation was positively associated with time to recovery of under-five admitted pneumonia patients.

Model comparison: The multivariate longitudinal sub-model was consistent with the results from the multivariate longitudinal analysis of RR, PR and oxygen saturation. The differences in magnitudes of the parameter estimates were negligible and there were some parameter difference in terms of statistical significance in separate MV longitudinal and separate survival model. But, longitudinal sub-model had narrow confidence interval which indicates that standard error is small for all significant predictors as compared to separate model in MV longitudinal and survival model. When evaluating the overall performance of both the separate and joint models in terms of model parsimonious and goodness of fit, the joint model was preferred as it has smaller standard error than the separate model. This result also supports the study done by [25, 26].

As Table 4 revealed, under MV joint model, estimate of the association parameters in the survival sub model was significantly different from zero (γ_1 = − 0.297, γ_2 = − 0.121 and γ_3 = 0.5452), this indicates that three longitudinal outcomes were correlated with time to recovery of under-five admitted pneumonia patients supported by [27–29], stats that the longitudinal and survival data are correlated. The joint model was more parsimonious fit than the separate model. Therefore, the joint model found preferable and parsimonious to fit the data better than the separate one [24] when the association parameter of the joint model is significant. Therefore, the final model for this study was joint model of MLMM and cox PH model.

## Discussion

The general objective of this study was identifying the determinant factors jointly affecting longitudinal measures of pneumonia (RR, PR and oxygen saturation) and time to recovery of under-five admitted pneumonia patients at FHRH, Bahir Dar, Ethiopia and the discussion was made based on results of Table 4.

The result reveals that about 89.1% of under-five patients were recovered from pneumonia with a median time of 72 hours (3 days) which took shorter recovery time compared to results of the study done by [2, 16, 30], whereas it is longer recovery time compared to results of the study done by [18, 31, 32]. The difference can be due to the difference in explanatory that we used, type of hospital etc. Age has significant effect on the two longitudinal measures of pneumonia (RR and PR), but had no information about longitudinal measures of oxygen saturation. When age of patients increase, the RR and PR measures decrease for under-five admitted pneumonia patients. This indicates that, lower level of pneumonia are found for increased age of patients. This was in line with results of the study conducted using nonlinear mixed model by [16]. Unlikely, using binomial logistic regression [33] found that age had no significant effect on measures of pneumonia. This requires further investigation to reach a decision in the effects of age on CAP.

Urban residency significantly increases the average values of RR and PR, whereas it decreases the average values of oxygen saturation of under-five admitted pneumonia patients. This indicates that, urban residency was significantly associated with the risk of pneumonia. This contradicts with results of the study done by [33] using binomial logistic regression. Based on the two contradicted ideas, we can suggest that in our country most of the people living in urban area have not their own living house and they live within a crowded room by using as living room and cooking room which is difficult to treat children and to gain fresh air. This makes children to be highly vulnerable to pneumonia compared to patients from rural residence. Smoking exposure increases the average values of RR and PR again it lowers the average oxygen saturation. This shows, smoking exposure related with increased level of pneumonia. This also agreed with results of the study conducted by [33] using binomial logistic regression for longitudinal data.

Having literate mothers increases the average oxygen saturation of under-five admitted pneumonia patients. This consides with results of the study conducted by [16]. Cooking food inside the living room lowers the average values of oxygen saturation which relates with high risk of pneumonia. As the follow-up time goes, the average values of RR and PR decreases, whereas the average values of oxygen saturation increases through visit time for under-five admitted pneumonia patients, which indicates effectiveness of treatment to lower pneumonia. In the survival sub model; Variables of urban residence, feeding exclusive breast within 6 months, first birth, non-danger sign and severity were significantly associated with recovery time of under-five admitted pneumonia patients. This was consistent with results of the study conducted by [31]. Increasing age of mothers increases the chance of experiencing the event of recovery (*p*-value = 0.026). This consides with results of the study conducted by [32]. The difference in the degree of significance may come from the difference in the variables as well as the model we used.

Exclusive breast feeding with in the first 6 months of life increases child survival by reducing the length of hospital stay. This supports results of the study done by [32]. The association parameters were significant indicates the significance of relationship between longitudinal measures of pneumonia (RR, PR and oxygen saturation) and time to recovery of under-five admitted pneumonia patients. This is in line with results of [34, 35]. Higher values of average RR and PR as well as lower values of average oxygen saturation were related with longer recovery time (high risk of pneumonia). This was consistent with results of the studies done by [18, 32].

## Conclusion

In this study, a joint model of multivariate longitudinal changes of respiratory rate, pulse rate and oxygen saturation with time to recovery of under-five admitted pneumonia patients was discussed. Out of the total sampled pneumonia patients 90 (89.1%) were recovered from pneumonia and the median recovery time was 72 hours. When evaluating the overall performance of both the separate (MLMM and cox PH model) and joint model in terms of model parsimonious, goodness of fit and the statistical significance of association parameters, the joint model performs better than the separate models. As a result, we concluded that the joint model was preferred for simultaneous analyses of repeated measurement and survival data. From results of the study, we can conclude that patients from urban area, borned at the first birth, having comorbid status, age of mother, exclusive breast feeding and having sever pneumonia have high levels of respiratory rate and pulse rate, whereas lower levels of oxygen saturation and which increases the risk of pneumonia. Patients with high levels of respiratory rate and pulse rate as well as low values of oxygen saturation requires longer recovery time of under-five admitted pneumonia patients. To improve child survival, the health professionals and community should be responsible for post ponding child birth and marriage.

## Data Availability

The datasets analyzed during the current study are available from the corresponding author upon reasonable request.

## Abbreviations

AIC: Acquired Immune Deficiency Syndrome
CAP: Community Acquired Pneumonia
CI: Confidence Interval
UDHS: Uganda Demographic and Health Survey
FHRH: Felege Hiwot Referral Hospital
LMM: Linear mixed model
MLMM: Multivariate Linear Mixed Model
MV: Multivariate
PH: Proportional Hazard
PR: Pulse Rate
RR: Respiratory Rate
S.E: Standard Error
SPSS: Statistical Package for Social Science
TCS: Time to Clinical Stability
UNICEF: United Nations International and Children’s Emergency Fund
WHO: World Health Organization
